# The usefulness of infrared spectroscopy and X-ray powder diffraction in the analysis of falsified, illegal, and medicinal products

**DOI:** 10.3389/fchem.2025.1536209

**Published:** 2025-02-19

**Authors:** Anna Mocarska, Karolina Piorunska, Jan K. Maurin, Agata Blazewicz

**Affiliations:** Falsified Medicines and Medical Devices Department, National Medicines Institute, Warsaw, Poland

**Keywords:** falsified medicinal products, illegal substances, API, analytical chemistry, FTIR, ATR, XRPD

## Abstract

One way to combat the black pharmaceutical market is to exchange experience and knowledge among the laboratories involved in this fight. A beneficial approach is compiling application examples that demonstrate the development and growing potential of the two analytical techniques that are undoubtedly useful in investigating pharmacologically active ingredients found in products dangerous to consumers health and life. Attenuated total reflectance Fourier transform infrared spectroscopy and X-ray powder diffraction are nondestructive techniques substantial for examining evidence seized by the police, demanding minimal preparation of the sample. Importantly, they are among the few that do not negatively impact the environment because they do not require the production or disposal of chemical reagents or solvents, aligning with the principles of green chemistry. Both techniques provide consistent, reproducible results, essential for legal and scientific validity.

## 1 Introduction

Although law enforcement agencies and analysts worldwide have been fighting against drug falsification for years, the market for falsified medications and the illegal use of pharmacologically active ingredients continues to thrive. Many of these products are sold without prescriptions on unregulated platforms, including websites, markets, and sex shops, often bypassing product control by being labeled, for example, as “not for human consumption” or “for research use only”. This opens up opportunities for misuse, as these drugs may contain incorrect or harmful ingredients or exist in various polymorphic forms, posing health risks. In many cases, they do not contain any active pharmaceutical ingredients (API) and consist only of excipients such as lactose, starch, sucrose, or mannitol. Falsified products are typically produced in poorly equipped laboratories, resulting in the presence of residual solvents and impurities (Sansone et al., 2021). Despite investigations and arrests, new manufacturing sites and distribution networks continue to emerge. Rapid identification of the composition of these products is crucial for law enforcement to intervene quickly and prevent the spread of these dangerous substances.

The structural similarities, physicochemical properties, and multicomponent matrices of these products pose substantial analytical challenges. Consequently, various techniques have been employed to determine the composition of falsified products. Common methods include chromatography, spectroscopy, titrimetry, electrochemistry, electrophoresis, nuclear magnetic resonance (NMR), and liquid chromatography combined with mass spectrometry (LC-MS) (Rao et al., 2023; Rebiere et al., 2017). High-performance liquid chromatography and ultraviolet-visible spectrophotometry are popular for their speed and precision. Titrimetric methods, such as potentiometric titration, and electrochemical techniques, such as voltammetry, are also widely used. NMR spectroscopy provides detailed molecular information (Keizers et al., 2019), while LC-MS is preferred for comprehensive drug assays and analysis of impurities and degradation products. Nondestructive methods, such as attenuated total reflectance-Fourier transform infrared spectroscopy (ATR-FTIR) and X-ray powder diffraction (XRPD), allow the identification of components that are discarded by other techniques during dissolution or filtration processes, which is an important aspect, especially in the case of excipients. This mini-review aims to display potential of both nondestructive techniques in the field of forensic science. While they share similarities, crucial differences make them complementary, enabling a comprehensive analysis of samples (Table 1).

**TABLE 1:** Similarities and differences between ATR-FTIR and XRPD in the analysis of suspected samples.

Attenuated total reflectance infrared spectroscopy (ATR-FTIR) European Pharmacopoeia (2021), Song et al. (2020)	X-ray powder diffraction (XRPD) European Pharmacopoeia (2022), Chauhan and Chauhan (2014)
Non-destructive	
Minimal preparation needed	
Identification of salts and polymorphs	
Access to databases or reference materials is necessary	
Provides an absorption spectrum	Provides a diffraction pattern
Suitable for solid samples, liquids, gases	Suitable for solid samples
Analysis of amorphous or crystalline sample components	Analysis of crystalline sample components
Smaller sample quantity needed (a few milligrams)	Larger sample quantity needed (several hundred milligrams)
Identification of molecular structure, functional groups, bonding	Identification of crystalline structure
Short analysis time (several dozen seconds)	Longer analysis time (several dozen minutes)

## 2 Usefulness and potential of the infrared (IR) technique in pharmaceutical analysis.

### 2.1 Basic properties of the IR technique and possible applications

IR spectroscopy involves changes in molecular energy due to absorbed IR radiation, causing vibrational and rotational motions. Absorbed radiation increases molecular energy, resulting in a spectrum with signals or bands. The position, intensity, and shape of these absorption bands are key characteristics. These bands correspond to the functional groups in the compound, as specific groups absorb IR radiation at defined frequencies, known as group frequencies, leading to characteristic vibrations. ATR is an IR measurement mode in which a sample is applied to a high-refractive-index crystal. Key phenomena include the reflection of the electromagnetic wave at the boundary between two media and its penetration into the lower optical density medium (European Pharmacopoeia, 2021). This technique allows for direct measurement of solid and liquid samples in less than a minute.

IR spectroscopy can analyze both mixtures and individual substances. The spectrum of a mixture is distinctive and confirms product authenticity, making it useful for examining suspected falsified products. Another purpose is to monitor changes within mixtures, such as confirming synthesis progress, decomposition, or surface adsorption. IR spectroscopy can identify different polymorphic forms of the same substance. The ATR mode is applicable for analyzing thick samples with strong absorption, as well as multilayered surface coatings and coatings on solid bodies. This technique relies on durable and chemically resistant crystals suitable for powders, solids, and even liquid samples by applying a single droplet (e.g., viscous liquids, biological materials, and aqueous solutions) (Song et al., 2020).

### 2.2 Application of ATR-FTIR technique in the analysis of falsified and substandard drugs

**FIGURE 1:**
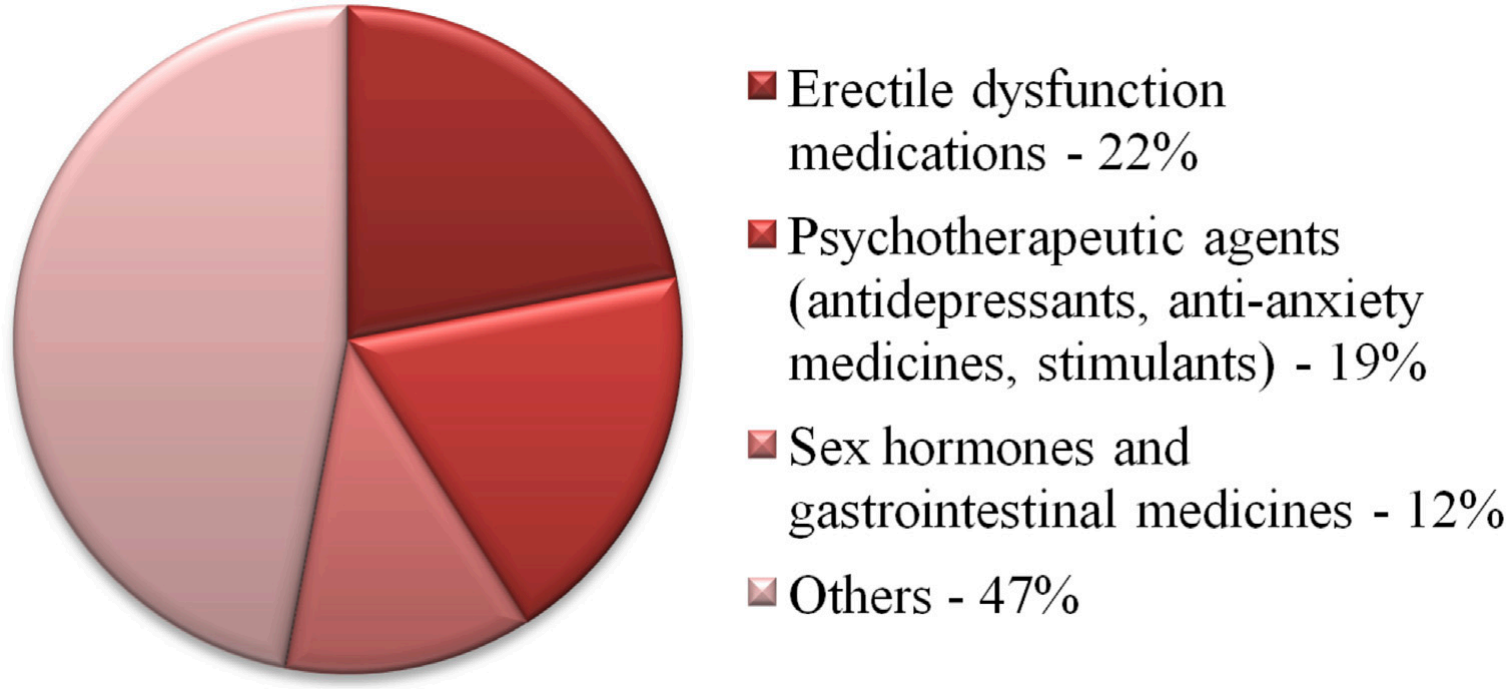
Distribution of drugs into pharmacological groups of products collected during Interpol’s global Pangea XVI 2023 campaign [3].

The most frequently falsified group of drugs are those used to treat erectile dysfunction (Figure 1) (Interpol, 2023). These data are reflected in the popularity of ATR-FTIR applications for testing this group of products. In a London-based study, three purported herbal supplements for men’s sexual performance were purchased from an e-commerce platform (Ho et al., 2022). Although these products were advertised as containing only herbal ingredients, sildenafil citrate was also detected in each. This was confirmed using several techniques, including ATR-FTIR. Measurements performed using this technique confirmed the presence of bands characteristic of sildenafil citrate, corresponding to N-H stretching, N-H bending, S=O symmetrical and asymmetric stretching, C–N stretching. Two of the products were unregistered generic tablets and one was a falsified medical product.

R.S. Ortiz et al. demonstrated the application of this technique using a diamond crystal for the rapid identification of falsified medicinal products containing phosphodiesterase type 5 inhibitors (Ortiz et al., 2013). The results revealed similarities between the mixtures used to prepare various counterfeit products. Similar findings were reported in a study of the same products on samples collected by law enforcement agencies in Poland (Piorunska-Sedlak and Stypulkowska, 2021). ATR-FTIR can also be used as an auxiliary technique accompanying the main research method. Products obtained from questionable online pharmacies in the Czech Republic were examined using Raman spectroscopy and mapping, but the results of identifying the qualitative composition were additionally confirmed by measurements in infrared spectroscopy (Spalovska et al., 2021).

In falsified drugs, active ingredients often originate from illegal sources. These ingredients may differ in efficacy from the original if they are in a different salt form or may pose additional risks if they contain impurities. Even a negative result of a test confirming the identity of an API without the possibility of identification due to database limitations may constitute valuable information. P.H.J. Keizers et al. after excluding the presence of sildenafil citrate by ATR-FTIR and confirming the presence of sildenafil by LC-MS decided to take the next step. They used several additional advanced techniques, including high-resolution tandem mass spectrometry, Raman microscopy, melting point analysis, and NMR spectroscopy, to demonstrate that sildenafil in the form of a mesylate, instead of citrate, can be found as an active ingredient in the illegal market (Keizers et al., 2016).

ATR-FTIR data can be further processed using numerous computational and chemometric methods. By applying singular value decomposition to calculate the wavenumber importance index, Brito et al. proposed a novel method for wavenumber selection (Brito et al., 2020). This method aimed to classify authentic and falsified drugs containing sildenafil and tadalafil citrate. A combination of these types of proceedings was also presented by other researchers (Mittal et al., 2021). Examination and analysis of results from over 400 antibiotic samples led to the development of a classification method aimed at detecting and identifying specific solid dosage forms of antibiotics. The combination of data recorded using the attenuated total reflection technique with the partial least squares–discriminant analysis method can be a useful tool for distinguishing between less and more sophisticated falsified Durateston^®^ products (Brazilian product containing an anabolic steroid) in a study of 96 samples (Neves et al., 2017).

IR spectroscopy can be used not only for qualitative analysis but also for quantitative analysis. A well-designed method can differentiate between substandard and falsified products. Researchers in the United Kingdom developed such a method using preparations containing paracetamol as an example (Lawson et al., 2018).

Not only synthetic drugs but also herbal medicines are subject to falsification. An Indonesian study presented an approach for the quantification and classification of herbal medicine products adulterated with synthetic drugs (prednisone and metamizole) by combining ATR-FTIR with multivariate calibration and discriminant analysis (Fatmarahmi et al., 2022).

The analysis of falsified drugs does not have to focus solely on the form of the active ingredients. Drug falsification also involves packaging, which can be used to assess the authenticity. M.R. Bin Salim et al. utilized this approach to study the blister packaging materials of paracetamol samples (Bin Salim et al., 2021). In another packaging study, a technique was used for the chemical analysis of vial label paper, flip-off caps, and cello tape on vial labels and stoppers (Degardin al., 2019). Additional techniques, such as Raman spectroscopy and microscopy, optical microscopy, and X-ray fluorescence spectroscopy, have also been employed. The combination of these tools enabled the examination of 31 falsified vials and revealed unexpected connections between counterfeits, which may be useful for supporting ongoing investigations.

### 2.3 Application of ATR-FTIR technique in analyzing pharmacologically active ingredients in other products

IR spectroscopic identification methods are applied not only to study medicinal products but also to analyze other substances that affect the human body, including new psychoactive substances (NPS), narcotic, and psychotropic substances.

In Poland, IR spectral analysis has been applied for the rapid differentiation of structural isomers among NPS (Piorunska-Sedlak and Stypulkowska, 2020). The combination of ATR-FTIR technique and computational methods has broad applications in analyzing various other substances. J. Hughes et al. described method for the rapid identification and quantification during police operations and seizures (Hughes et al., 2013). A thoroughly developed and validated method can be used as an inexpensive, fast, and mobile alternative to expensive and labor-intensive processes. Darie et al. analyzed spectra to classify various drugs of abuse, including hallucinogens, cannabinoids, and opioids, using machine learning models, such as support vector machines and eXtreme Gradient Boosting (Darie et al., 2023). An interesting application of spectral data involves the identification and selection of compounds that can be used as surrogates for opioids in the development and training of spectroscopic sensors. Compounds such as heroin, fentanyl, and carfentanil have been studied to enhance the safety of drug enforcement officer training (Daniels et al., 2022). Chemometric models also enable the use of spectroscopic data to determine the origin of drugs based on impurity profiles, characteristic of specific synthetic pathways. McKeown et al. described the application of the orthogonal partial least squares-discriminant analysis model to the fentanyl precursors N-phenethyl-4-piperidone and 4-anilino-N-phenethylpiperidine (McKeown et al., 2023). The combination of experimental and computational approaches in the analysis of atomic locality for selected vibrational models and well-known compounds, such as variously substituted piperidine, facilitates the detection of novel derivatives in illicit drug-related samples and supports the identification of fentanyl and its analogs, including salts, in environmental samples (Shan et al., 2022). Due to the sensitivity of this technique to polymorphisms, it is often used in studies of APIs. Combined with Raman spectroscopy and computational methods, this technique has proven useful for identifying different polymorphic forms of pyrazinamide in mixtures (Zhou et al., 2024).

A different case was reported by J. Coelho Neto et al. They examined colorful tablets sold as ecstasy (MDMA) (3,4-methylenedioxymethamphetamine) and found no traces of the declared MDMA (Neto et al., 2018). However, they detected spectral bands characteristic of sildenafil. The tablets were repainted genuine Brazilian products for the treatment of erectile dysfunction.

Another application was shown in quantitative analysis. Ramsay et al. described the role of ATR-FTIR as part of a multi-technology approach used to determine fentanyl in complex mixtures containing caffeine and fentanyl in illicit opioids (Ramsay et al., 2021).

The examples mentioned show that ATR-FTIR and Raman spectroscopy are often used together. They are complementary techniques in modern vibrational spectroscopy, as they are based on different phenomena: IR absorption and Raman scattering. Raman spectroscopy detects vibrational modes that involve changes in molecular polarizability, making it effective for analyzing non-polar bonds, while IR spectroscopy identifies modes associated with changes in dipole moments, making it sensitive to polar functional groups. Therefore, together they provide a more comprehensive molecular characterization of pharmaceutical compounds, excipients, and falsified drugs by capturing different vibrational features governed by distinct selection rules (Muro et al., 2015).

## 3 Usefulness and potential of the XRPD technique in pharmaceutical analysis

### 3.1 Basic properties of the XRPD technique and possible applications

Each crystalline substance is characterized by a distinct crystal system composed of regularly repeating structural elements, such as atoms, ions, and molecules. These elements form lattice planes, whose arrangement can act as a diffraction grating for radiation. The diffraction of radiation, in this case, X-rays, arises from interactions with the electron clouds of atoms. Depending on the specific arrangement of the atoms, the incident radiation can be either enhanced or diminished in the crystal lattice. The amplification condition requires that the path difference between two diffracted X-ray waves must be an integral multiple of the wavelength, a principle described by Bragg’s law (European Pharmacopoeia, 2022). When this requirement is fulfilled, strongly reflected X-rays are produced at specific angles and recorded as a function of the angle to produce diffraction patterns. The positions and intensities of the peaks can be analyzed because they are characteristic of different crystalline substances.

XRPD is a highly effective analytical technique with a long history of use. For years, it has been used to analyze and characterize crystalline structures, providing information on both active pharmaceutical ingredients and excipients. Other advantageous application of the method is detection of changes in polymorphic structure of the substance, as indicated by numerous research papers (Thakral et al., 2018; Spiliopoulou et al., 2020; Rodríguez et al., 2021). This is crucial because different polymorphic forms can have different properties that affect the bioavailability, stability, and manufacturing of API (Ainurofiq et al., 2020). Differences in polymorphic forms can be clear indicators of falsified products because legitimate drugs are manufactured under strict controls to maintain consistency in their crystalline forms. Moreover, this technique can detect variations in excipients and coatings, thereby distinguishing between genuine and counterfeit medicines. As a nondestructive analytical tool, it supports the pharmaceutical industry in quality control, regulatory compliance, and the development of novel therapeutic agents, thereby enhancing overall drug discovery and development.

### 3.2 Application of XRPD technique in the analysis of falsified and substandard drugs

XRPD offers substantial development potential for the detection and prevention of drug falsification, which is an area of growing concern in pharmaceutical regulations and consumer safety. This method provides a highly accurate means of verifying the authenticity of various medications, especially when the reference diffraction pattern of the original product is available.

XRPD is a technique used to analyze drugs for erectile dysfunction. Such investigations were evaluated in Poland, covering the topic of falsification of the well-known medication Viagra^®^ containing sildenafil citrate. Substantial differences in diffraction patterns have been observed between authentic and falsified products (Piorunska-Sedlak and Stypulkowska, 2021; Maurin et al., 2007). Moreover, E Deconinck et al. employed chemometric models to distinguish between tadalafil and sildenafil citrate samples from different manufacturers, which can help in determining the API sources (Deconinck et al., 2022; 2023). The aforementioned London-based study also provided valuable insights into the physical form of the active ingredient, its phase transitions, and the potential impact on product performance through the use of simultaneous differential scanning calorimetry and XRPD techniques (Ho et al., 2022). Similar investigations have been carried out on other medications, for example, on the anti-ulcer medication omeprazole (Rebiere et al., 2022), and also to differentiate between genuine and counterfeit medicines claiming to contain acetylsalicylic acid and ascorbic acid (Jendrzejewska et al., 2018). These examples illustrate the broad applicability of this technique in pharmaceutical authentication.

To increase revenue, falsified drug producers frequently use cheaper alternatives for both active pharmaceutical ingredients and excipients. These alternatives may be of inferior quality, ineffective, or even hazardous, posing serious health risks to consumers. Research on drugs containing paracetamol has shown that XRPD can effectively reveal quality inconsistencies between different pharmaceutical products with the same active ingredients and distinguish polymorphic forms of paracetamol in different drug formulations (Oloyede et al., 2023; Jendrzejewska et al., 2020). This is particularly important because legitimate pharmaceuticals are produced under tightly controlled conditions to ensure that the crystalline form of the active ingredient remains consistent across all batches. Any deviation in these forms may suggest that the product is falsified or substandard. The ability of XRPD to identify these subtle yet critical differences makes it a valuable tool in fighting drug falsification.

### 3.3 Application of XRPD in the analysis of pharmacologically active ingredients in other products

The application of XRPD extends beyond the identification of falsified drugs. It can be applied not only to final products but also during the pre-approval drug studies. Stability studies are pivotal in the pharmaceutical industry for estimating the long-term behavior of substances. Environmental conditions such as temperature (Kumar and Jha, 2017), light exposure, and humidity (Ng et al., 2022) can cause changes in the crystalline structure of APIs and excipients, thereby affecting the stability, efficacy, and safety of medicines. Phase transitions, amorphization, and recrystallization are phenomena that may occur (Jojart-Laczkovich et al., 2016). Understanding these changes is essential for establishing proper storage conditions and identifying degradation products or impurities, which can optimize formulations. Amorphous drugs often exhibit higher solubility and dissolution rates than their crystalline counterparts (Kapoor et al., 2023).

Therefore, studies using XRPD to monitor changes in medicines are very popular, leading to many applications, such as in the field of co-amorphous formulations. M. Ruponen et al. analyzed furosemide with arginine and P-glycoprotein inhibitor drugs (Ruponen et al., 2021), while other scientists evaluated the stability of amorphous olanzapine and its co-amorphous form with saccharin (da Costa et al., 2022). There was also a Danish research conducted on co-amorphous systems consisting of naproxen, arginine, and lysine (Wostry et al., 2020). An investigation into diabetes treatment in this area showed that mixing poorly soluble nateglinide with metformin hydrochloride enhanced the dissolution rate of nateglinide. The amorphization and stabilization of this form in the mixture were confirmed, leading to increased drug release and the potential for improved therapy in patients with diabetes (Wairkar and Gaud, 2016). Other studies focused on developing inclusion complexes of meloxicam with β-cyclodextrin-based nanosponges that should enhance solubility, stability, and prolong drug release. The crystalline form of meloxicam converts into an amorphous state through complexation with nanosponges, which is a new approach in terms of drug release (Shende et al., 2015). Similar studies were conducted in Poland on inclusion complexes of ketoprofen (Betlejewska-Kielak et al., 2021) and flurbiprofen (Betlejewska-Kielak et al., 2023) with β-cyclodextrin, proving the formation of a complex.

Determining which crystalline form of the compound occurs in a given drug is crucial. In this regard, XRPD technique experiments were carried out with segesterone acetate, a drug used as a contraceptive (Aragon et al., 2021), flavonoids such as taxifolin (Terekhov et al., 2020), and antiepileptic drug levetiracetam (Xu et al., 2015). In a previously mentioned study of different polymorphic forms of pyrazinamide, XRPD was used for identification (Zhou et al., 2024).

Another interesting topic that has attracted increasing attention is the production and distribution of NPS. These substances are typically sold online and marketed as fertilizers, bath salts (Altun and Cok, 2020; Riley et al., 2020), and research chemicals (Hohmann et al., 2014; Thornton et al., 2019). XRPD can be a useful tool for identifying NPS not only in the form of pure substances but also in street mixtures containing both organic and inorganic impurities (Jurasek et al., 2019).

## 4 Discussion and future outlook

Attenuated total reflectance- Fourier transform infrared spectroscopy and X-ray powder diffraction are nondestructive analytical techniques that allow direct measurement of samples with minimal preparation, thus preserving their original composition. Both techniques are noninvasive, simple, and reproducible, enabling the identification of both the main and auxiliary components. The combination of the techniques enables the rapid and accurate identification of contaminants or falsified substances, including salts, co-crystals, mixtures, and various polymorphic forms (Table 2), although both techniques require access to databases or reference materials. Therefore, they are well suited for rapid screening tests and identification of known compounds registered in databases. Negative results of database searches direct further research towards the use of other techniques for identifying unknown substances or detecting low concentrations of compounds, such as LC-MS or NMR. Whereas ATR-FTIR has long been established for its speed and simplicity, the importance of XRPD has grown due to advances in miniaturization and faster analysis. They play relevant roles in confirming the identity and authenticity of products, highlighting their importance in ensuring product safety and quality across regulatory and industrial settings.

**TABLE 2:** Applications of ATR-FTIR and XRPD techniques in the study of products and compounds with pharmacological activity.

Technique	Subject of study		References
	Category	Reason for using/substance	
Falsified and substandard			
ATR-FTIR	Falsified herbal supplements	Men’s sexual performance	Ho et al. (2022)
	Authentic and falsified medicinal products	Blister packaging materials	Bin Salim et al. (2021), Degardinet al. (2019)
	Authentic/falsified medicinal products	Pain relievers (paracetamol)	Lawson et al. (2018)
	Falsified medicinal products	Erectile dysfunction disorder drugs (Phosphodiesterase-5 Inhibitors- PDE5-i)	Ortiz et al. (2013), Piorunska-Sedlak and Stypulkowska (2021), Spalovska et al. (2021)
	Falsified API		Keizers et al. (2016)
ATR-FTIR/chemometric	Authentic/falsified medicinal products		Brito et al. (2020)
		An anabolic steroid	Neves et al. (2017)
	Medicinal products	Antibiotics	Mittal et al. (2021)
	Authentic and falsified herbal medicinal products	Pain relievers	Fatmarahmi et al. (2022)
XRPD	Falsified herbal supplements	Men’s sexual performance	Ho et al. (2022)
	Substandard medicinal products	Pain relievers (paracetamol)	Oloyede et al. (2023), Jendrzejewska et al. (2020)
	Falsified medicinal products	Acetylsalicylic acid and ascorbic acid	Jendrzejewska et al. (2018)
	Falsified medicinal products	Erectile dysfunction disorder drugs (Phosphodiesterase-5 Inhibitors- PDE5-i)	(Piorunska-Sedlak and Stypulkowska, 2021) Maurin et al. (2007)
XRPD/chemometric	Authentic/substandard/falsified API		(Deconinck et al., 2022; 2023)
		Omeprazole	Rebiere et al. (2022)
Illegal and others			
ATR-FTIR	Illegal products/authentic/falsified medicinal products	MDMA/PDE5-i	Neto et al. (2018)
	Illegal products	Heroin, fentanyl, and carfentanil surrogates	Daniels et al. (2022)
		New psychoactive substances- NPS	Piorunska-Sedlak and Stypulkowska (2020)
		Fentanyl	Ramsay et al. (2021)
ATR-FTIR/chemometric		New psychoactive substances- NPS	Darie et al. (2023)
		Methamphetamine	Hughes et al. (2013)
		Fentanyl precursors	McKeown et al. (2023)
		Fentanyl and analogs	Shan et al. (2022)
	API (polymorphic forms)	Pyrazinamide	Zhou et al. (2024)
XRPD	API (co-amorphous formulations)	Furosemide with arginine and P-glycoprotein inhibitor drugs	Ruponen et al. (2021)
		Olanzapine with saccharin	da Costa et al. (2022)
		Naproxen and arginine with lysine	Wostry et al. (2020)
		Nateglinide with metformin hydrochloride	Wairkar and Gaud (2016)
	API (inclusion complexes)	Meloxicam with β-cyclodextrin-based nanosponges	Shende et al. (2015)
		Ketoprofen with β-cyclodextrin	Betlejewska-Kielak et al. (2021)
		Flurbiprofen with β-cyclodextrin	Betlejewska-Kielak et al. (2023)
	API (polymorphic forms)	Segesterone acetate	Aragon et al. (2021)
		Taxifolin	Terekhov et al. (2020)
		Levetiracetam	Xu et al. (2015)
	Illegal products	New psychoactive substances- NPS	Jurasek et al. (2019)
XRPD/chemometric	API (polymorphic forms)	Pyrazinamide	Zhou et al. (2024)
